# A Rare Case of Aneurysmal Bone Cyst in the Paranasal Sinus

**Published:** 2015-09

**Authors:** Seyyed Mostafa Hashemi, Mitra Heidarpour, Afrooz Eshaghian, Peyman Ansari, Maryam Sadat Hashemi, Maryam Yaghoobi, Sohrab Barati

**Affiliations:** 1 *Department of Otorhinolaryngology, Kashani Hospital, Isfahan University of Medical Sciences, Isfahan, Iran.*; 2 *Department of Pathology, Kashani Hospital, Isfahan University of Medical Sciences, Isfahan, Iran.*; 3 *Department of Radiology, Kashani University of Medical Sciences, Isfahan, Iran.*; 4 *General Practitioner, Isfahan University of Medical Sciences, Isfahan, Iran.*

**Keywords:** Aneurysmal Bone Cysts (ABC), Angiofibroma.

## Abstract

**Introduction:**

Aneurysmal Bone cysts (ABC) are extremely rare in the head and neck region and even rarer in sinuses. ABC is a benign multicystic mass that is locally-destructive and rapidly expandable. Hemorrhagic fluid content (like in this case) and septated appearance are the characteristic feature of ABC. Established treatment options for ABCs include sclerotherapy, embolization, radiotherapy, simple curettage, surgical excision, or a combination of methods.

**Case Report:**

In this article, a 5 year-old boy with a recurrent nasal mass is presented. The patient was finally diagnosed with this rare entity: ABC of the paranasal sinuses. The patient was treated through complete surgical removal.

**Conclusion:**

ABC can be considered as a rare differential diagnosis of recurrent nasal hemorrhagic mass in a pediatric population.

## Introduction

Aneurysmal Bone cysts (ABC) are vascular tumors that show bony expansion. ABC rarely occurs in the head and neck region and even less so in the maxilla and sinuses (1,2). ABC is considered as a secondary tumor to certain pathological bone lesions (3). Its prevalence is about 1.4% of all bone tumors and about 3% in the cranium. It is most commonly found in patients less than 20 years of age in both genders (4). Radiographical findings contain an eccentric lytic lesion with an expanded remodeled "blown-out" or "ballooned" bony contour of the affected bone, with a delicate trabeculated appearance, and fluid-filled spaces on CT scans and MRI (3). 

In this article, a 5 year-old boy with a recurrent nasal mass is presented. The patient was finally diagnosed with this rare entity: ABC of the paranasal sinuses. 

## Case Report

The patient was a 5-year-old boy who was presented to our clinic with symptoms of nasal stiffness, snoring, and right nostril purulent rhinorrhea since 3 months. His parents also explained that he had progressive proptosis of the right eye during this time. He had no pain, visual disturbances, or rhinorrhagia. Three days before admission, he had a discharge from his eye. Adenoidectomy had been done one month ago, but his symptoms had partially recovered; and purulent rhinorrhea remained after his previous surgery.

He complained about repeating common colds in the last eight months, which were treated by antibiotics and symptomatic medication; however, their effect did not completely erase the symptoms. He had no previous medical history.

Nasal examination revealed a right and left firm nasal mass, extending to the inferior conchae with a non-bloody nasal discharge. Ophthalmological examination revealed limited lateral gaze on the right eye. His laboratory data contained: WBC: 8000 (48.1% neut, 41.5%lymph), Hemoglobine: 10.8, Hematocrite: 34.4, MCV: 75.6, MCH: 23.7, MCHC: 31.4, Platelet: 371000, PT: 13, PTT: 31, INR:1.

**Fig 1:**
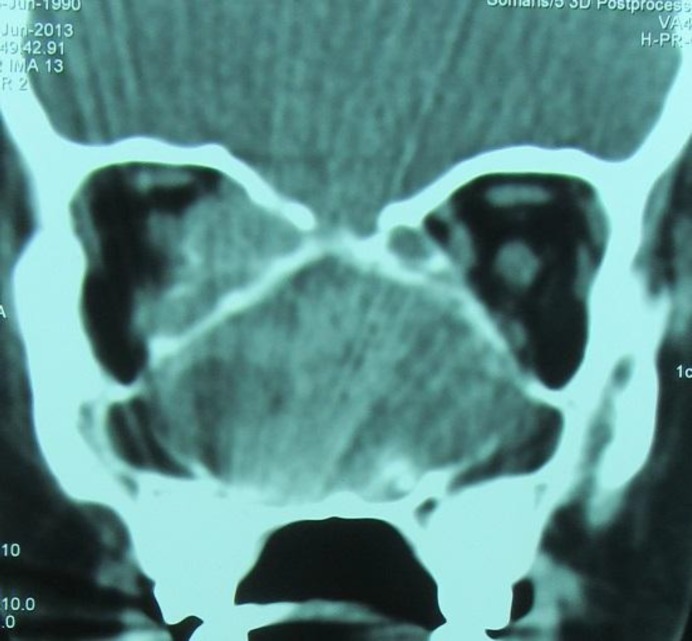
Coronal CT scan of the paranasal sinuses: large well defined heterodense mass lesion (5-6cm) in the right nasal cavity, extending to the nasopharynx and involving the entire sphenoid sinus

A spiral CT scan, without contrast, of the orbit was performed. A large well-defined heterogeneous mass lesion (5-6cm) in the right nasal cavity, extending to the nasopharynx and involving the entire sphenoid sinus, was observed. The lesion showed hypodense foci and remodeling of bones was seen. The mass compressed the maxillary sinuses (bowing of lateroposterior wall of the antral sinuses and medial wall of the orbit was noted) resulting in a compressive effect on the right inferior muscle, medial rectus muscle, and optic nerve. Obstruction of the drainage of the antral, ethmoid, and sphenoid sinuses; in addition to the right nasolacrimal duct was seen. No destruction of bone was seen. There was a dense shell around the mass, which seemed to be calcium. The lesion had a sinonasal origin. Erosion of the sella turcica was seen; but the mass did not extend to the brain. These findings suggested a slow growing mass. Radiological findings suggested ossifying fibroma or angiofibroma (Fig.1).

**Fig 2:**
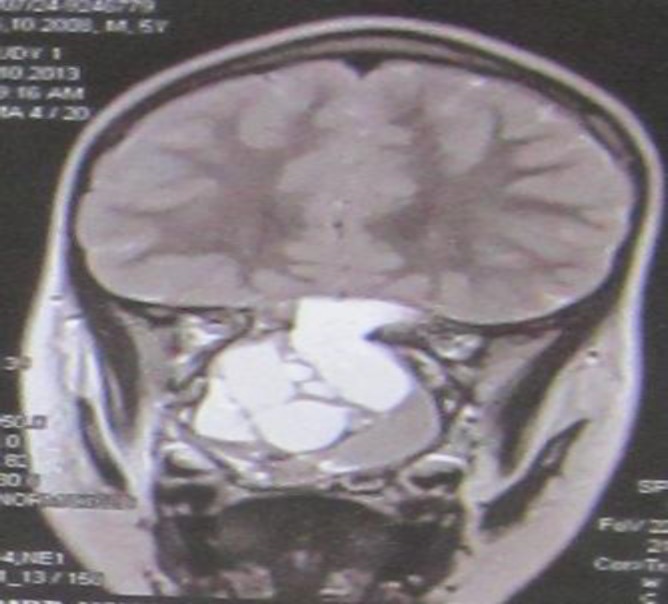
Fluid-attenuated inversion recovery (TR: 9000, TE: 2500) weighted coronal image of sinus: an expansile non-homogenous mass lesion with multiple and variable sizes of cystic lesions between solid parts

MRI of the nasal cavity with Gadolinium was done. It revealed that the nasal cavity had been replaced by an expansile non-homogenous mass lesion. Cystic lesions (of multiple and variable size) between solid parts were evident. No intracranial extension was seen. The lesions extended into the right orbit. Following administration of contrast media enhances in solid part. MRI suggested an expansile multiple cystic lesion with a bony origin such as Aneurysmal Bone cyst, Esthesioneuroblastoma, Hystyocytosis or parasitic cyst (Fig.2). 

Through endoscopic approach, a midline mass extending from the posterior septum to the lateral nasal wall was observed. After opening of the nasal septal mucosa, through shaver necrosis, the tumor was drained. In the right side, the right and left ethmoidal sinus was opened and cleared until the medial wall of the orbit. In the nasopharynx, the tumor was located anterosuperiorly to the skull base, and was cleaned. Except for the mid-part of the fovea ethmoidalis, other parts of the skull base were free of tumor. Fovea ethmoidalis had bony erosion and was cleaned. After surgery, the patient had a decrease in Hemoglobin level from 108 to 5.9, so 350 cc packed cell were infused. The patient was in ICU for two nights and was then discharged on the third day.

Pathological study showed macros- copically soft pink to yellow gray tissues. During microscopic examination, there was an extensive vascular proliferation in a fibrosis context, with macrophages contained in hemocydrine and hemorrhage. The diagnosis of Angiofibroma was suggested. After five months, the patient was again referred with symptoms of proptosis, nasal stiffness, bloody purulent rhinorrhea, and vertigo. Visual acuity decreased and there was optic disc swelling in the left eye, and pallid optic disc after swelling in the right eye. 

**Fig 3:**
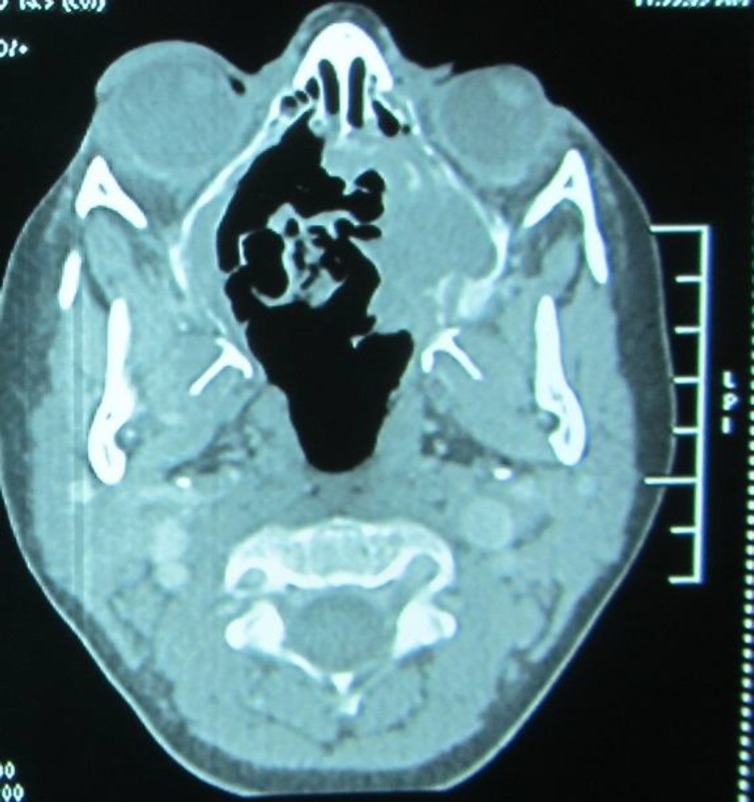
Axial CT scan of the paranasal sinus with contrast revealed an expansion in the posterior aspect of the ethmoidal and sphenoidal sinus

New facial CT scan was done and there was an expansion observed in the posterior aspect, from the ethmoidal until the sphenoidal sinus accompanied with soft tissue density. It caused anterior displacement of the posterior wall of the right maxillary sinus. Displacement of the medial wall of both the frontal bone and muscles to the lateral side was also seen. Aneurysmal Bone cyst, Burkitt lymphoma, Hystiocytosis were the suggested differential diagnosis. CT scan of abdomen, thorax, and neck were normal (Fig. 3).

An endoscopic surgery was done for the second time. There was a cystic mass, which was aspirated. The aspirated fluid was an old brown bloody liquid. The lateral wall of the mass was then excised, and the mass of the sphenoid and skull base was removed. Dura, skull base, and perineural spaces were cleaned from the tumor.

**Fig 4:**
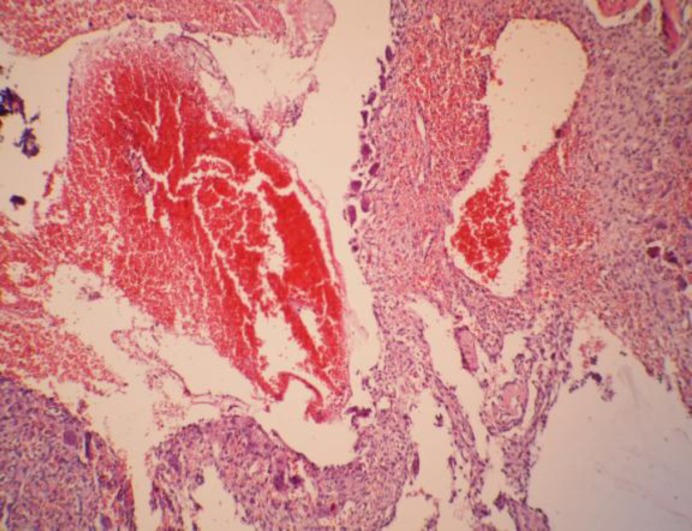
Histopathology of the lung (H&E stain, ×40). mesenchymal proliferation with bony and vascular extension

A new pathological study revealed diffuse mesenchymal proliferation, with long nuclei and moderate focal pleomorphism. There was bony and vascular extension (Fig.4).

Immunohistochemical study revealed Vimentin: positive, B Catenin: positive, CD117: positive, SMA: positive, Desmin: negative, CD34: negative. Once again, these finding suggested cellular angiofibroma. Due to the mismatch between clinical, radiological, and pathologic findings, the pathological slides were consulted with different pathologists. They reported proliferating stromal cells with intervening scattered multinucleated giant cells of osteoclastic type in some areas. The lesion is vascular and contains blood field spaces surrounded directly by stromal and giant cells. Deposition of osteoid is also present near the cavernous spaces. They reported Aneurysmal Bone cyst, nasal, and paranasal sinus involvement.

The patient was referred to a pediatric oncologist for further follow up. They evaluated the whole body to rule out metastases of sarcoma, and also evaluated the bone marrow. Bone marrow aspiration was normal. In the abdominal sonography, there were multiple para-aortic lymph nodes (maximum size was 4 mm). Other images revealed no metastases or any evidence of other tumors.

The patient was followed clinically for any signs or symptoms of recurrence. The patient has not had any problems so far (six months after surgery).

## Discussion

This article presents a rare case of ABC in the paranasal sinuses, which was diagnosed as angiofibroma during the first evaluation. The radiological and pathological findings suggested that this tumor, and surgical findings were compatible with ABC. ABCs can be primary or secondary to another skeletal lesion. Secondary ABCs may be related to fibrous dysplasia, giant cell tumors, chondroblastoma, chondromyxoid fibroma, non-ossifying fibroma, fibrous histiocytoma, osteoblastoma, and osteosarcoma (5). It can be considered as a primary or secondary tumor to angiofibroma; however, clinical behavior improves the diagnosis of ABC in this case.

Few cases of ABC in the sinuses were reported up to now (6-9). Skull and facial skeleton are involved in 3 to 6 % of cases (10).The mandible is the most common site in this region (11); and the maxilla is even more rare (2). ABS is very rare in turbinates (12).

Hemorrhagic fluid content (like in this case) and septated appearance is the characteristic feature of ABC (12). ABC is a benign multicystic mass that is locally-destructive and rapidly expandable (8).

ABCs have some characteristic features in CT and MRI. In CT images, erosion, thinning of cortex, and ridges in the bony walls, with 30% Fluid levels on bone, are seen. In MRI images, well-defined heterogeneous signal intensity lesion on T1, hypointense capsule, and a homogenous increase in signal intensity on T2 is observed. During angiograpy, the blood supply to these vascular lesions can occasionally reveal arteriovenous shunts (5). This case had typical CT and MRI findings, but it wasn’t considered as the first diagnosis because of the rarity of ABC in this region. Treatment options considered for ABCs include sclerotherapy, embolization, radiotherapy, simple curettage, surgical excision, or a combi- nation of methods. Surgical excision is the first treatment of choice, and there is a 10 to 30% recurrence rate (12). 

## Conclusion

Aneurysmal Bone cyst (ABC) is extremely rare in sinuses. It can be diagnosed by specific radiographical findings containing an eccentric lytic lesion with an expanded and remodeled "blown-out" or "ballooned" bony contour of the affected bone, in addition to a delicate trabeculated appearance, and fluid-filled spaces on CT scans and MRI. ABC is a benign multicystic mass that is locally-destructive and rapidly expandable. Even in childhood this should be considered as a differential diagnosis.
